# Endocannabinoid 2-arachidonoylglycerol is elevated in the coronary circulation during acute coronary syndrome

**DOI:** 10.1371/journal.pone.0227142

**Published:** 2019-12-30

**Authors:** Julian Jehle, Hanna Goerich, Laura Bindila, Beat Lutz, Georg Nickenig, Vedat Tiyerili

**Affiliations:** 1 Department of Internal Medicine II Cardiology, Pneumology, Angiology, University Hospital Bonn, Bonn, Germany; 2 Institute of Physiological Chemistry, University Medical Center of the Johannes Gutenberg University Mainz, Mainz, Germany; University of Bologna, ITALY

## Abstract

**Objectives:**

The endocannabinoid system modulates coronary circulatory function and atherogenesis. The two major endocannabinoids (eCB), 2-arachidonoylglycerol (2-AG) and *N*-arachidonoylethanolamide (AEA), are increased in venous blood from patients with coronary artery disease (CAD). However, given their short half-life and their autocrine/paracrine mechanism of action, eCB levels in venous blood samples might not reflect arterial or coronary eCB concentrations. The aim of this cross-sectional study was to identify the local concentration profile of eCB and to detect whether and how this concentration profile changes in CAD and NSTEMI versus patients without CAD.

**Methods and results:**

83 patients undergoing coronary angiography were included in this study. Patients were divided into three groups based on their definite diagnosis of a) no CAD, b) stable CAD, or c) non-ST-segment elevation myocardial infarction (NSTEMI). Blood was drawn from the arterial sheath and the aorta in all patients and additionally distal to the culprit coronary lesion in CAD- and NSTEMI patients. 2-AG levels varied significantly between patient groups and between the sites of blood extraction. The lowest levels were detected in patients without CAD; the highest 2-AG concentrations were detected in NSTEMI patients and in the coronary arteries. Peripheral 2-AG levels were significantly higher in NSTEMI patients (107.4 ± 28.4 pmol/ml) than in CAD- (17.4 ± 5.4 pmol/ml; p < 0.001), or no-CAD patients (23.9 ± 7.1 pmol/ml; p < 0.001). Moreover, coronary 2-AG levels were significantly higher in NSTEMI patients than in CAD patients (369.3 ± 57.2 pmol/ml vs. 240.1 ± 25.3 pmol/ml; p = 0.024).

**Conclusions:**

2-AG showed significant variability in arterial blood samples drawn from distinct locations. Possibly, lesional macrophages synthesise 2-AG locally, which thereby contributes to endothelial dysfunction and local inflammation.

## Introduction

Inflammation drives coronary artery disease at all stages from endothelial dysfunction, fatty streak, and foam cell formation to the thinning of the fibrous cap that precedes atherosclerotic plaque rupture and myocardial infarction [1]. The endocannabinoid system (ECS) modulates these inflammatory processes. The ECS consists of the cannabinoid receptors and their endogenous ligands, the two best studied of which are the endocannabinoids (eCB) *N*-arachidonoylethanolamide (AEA) and 2-arachidonoylglycerol (2-AG). Furthermore, the enzymes that synthesise and degrade the endocannabinoids are part of the ECS, as well as putative eCB transporters, which are supposed to enable facilitated diffusion but have not been cloned yet [2–5].

The ECS is deeply involved in the initiation and progression of coronary artery disease: first, eCB control endothelial relaxation and vascular tone as shown in human and murine studies [6–10]. Second, both major eCB, AEA and 2-AG have been demonstrated to promote vascular inflammation and atherogenesis in murine models [11–14]. Finally, 2-AG has been demonstrated to induce platelet activation and thrombus formation (reviewed by [15]). Most of these mechanisms can be attributed to the local activity of eCB via autocrine and paracrine signalling pathways [16]. However, most studies describing the local activity of eCB within the coronary arteries have attributed their findings to systemic eCB concentrations, measured from venous blood [6, 7, 17]. This major methodological flaw was first addressed by Maeda and coworkers. They measured endocannabinoid levels in blood samples drawn directly from the coronary arteries of 43 patients suffering from acute ST-elevation myocardial infarction (STEMI) and a small group of six patients with stable angina. In STEMI patients, Maeda et al. found the AEA concentration to be significantly elevated in the infarct-related coronary artery compared to the aortic root. Meanwhile, 2-AG was only detected in six out of 43 STEMI patients and neither eCB was detected in stable patients [18].

Contemporary liquid chromatography/multiple reaction monitoring (LC/MRM) analyses allow for the highly sensitive analysis of endocannabinoid concentrations, hence detection of minute amounts of molecular targets in biological matrices. Capitalising on advanced LC/MRM methodology, we sought to determine, whether AEA, 2-AG, palmitoylethanolamide (PEA), and arachidonic acid (AA) are synthesised locally in patients with coronary artery disease (CAD) and non-ST-segment elevation myocardial infarction (NSTEMI). The aim of this study was to identify the local concentration profile of endocannabinoids and arachidonic acid at distinct sites within the arterial and venous vasculature and to detect whether and how this concentration profile changes in CAD and NSTEMI versus patients without CAD.

## Materials and methods

### 2.1 Study protocol and study population

83 patients were included in this study: 15 of whom were diagnosed with NSTEMI, 48 suffered from coronary artery disease without unstable angina or elevated cardiac troponin levels, and 20 patients presented without significant stenotic coronary artery disease. NSTEMI was defined as an acute onset of persistent (>20 minutes) anginal pain at rest with elevated and dynamically changing troponin levels in the absence of persistent (>20 minutes) ST-segment elevations on 12-lead ECG, according to the current definition of myocardial infarction by the European Society of Cardiology [19]. Significant stenotic coronary artery disease was defined as narrowing of the lumen by at least 50% in the left main coronary artery or by at least 70% in one or more of the other major coronary arteries based on coronary angiogram, according to current guidelines [20]. It was assumed that patients who had undergone percutaneous coronary intervention (PCI) prior to this study had significant coronary artery disease. Exclusion criteria comprised acute infections, immunosuppressive therapy, preexisting autoimmune diseases, malignant diseases, and cardiogenic shock. Written informed consent was obtained from all patients prior to their inclusion in the study. The study protocol was approved by the local ethics committee (Ethikkommission an der Medizinischen Fakultät der Rheinischen Friedrich-Wilhelms-Universität Bonn) and is in accordance with the Declaration of Helsinki and its subsequent revisions. Ethical concerns were raised by the local ethics committee regarding the inclusion of STEMI patients, because these patients might not be able to provide written informed consent prior to percutaneous coronary intervention (PCI) in an emergency setting. Thus, STEMI patients could not be included in the present study.

An additional five patients, who underwent combined right- and left-heart catheterisation, were included for the assessment of eCB levels in the low-pressure system. These patients underwent combined right- and left-heart catheterisation for suspected coronary artery disease and suspected pulmonary hypertension. The majority of these patients (4/5) were diagnosed with group 2 pulmonary hypertension due to mitral regurgitation.

### 2.2 Blood sampling, processing, and storage

Blood was drawn from patients undergoing coronary angiography for suspected coronary artery disease. Blood extraction sites were the sheath, the aorta, and the poststenotic culprit coronary artery.

The choice of radial or femoral access was left up to discretion of the interventional cardiologist. Radial or femoral blood was drawn from the side port of the arterial sheath (Terumo 6F; Terumo, Tokyo, Japan). Aortic blood was drawn from the aortic sinus, proximal to the aortic valve, using a pigtail catheter (Boston Scientific, Marlborogh, USA). Coronary blood was drawn from the coronary artery that underwent angioplasty in CAD- and NSTEMI patients (i.e. the suspected culprit coronary artery). The stenosis was passed using a SAMURAI^™^ guidewire (Boston Scientific). Then a 1.5 mm /10 mm over-the-wire (OTW) balloon dilatation catheter (Boston Scientific) was advanced past the stenosis and the wire was removed before blood was drawn, prior to angioplasty.

The coronary blood sample was omitted in patients with purely diagnostic procedures who neither proceeded to PCI nor to fractional flow reserve- (FFR) or intravascular ultrasound (IVUS) measurements, because the extraction of blood via an OTW catheter would have necessitated systemic anticoagulation with heparin as well as the wiring of a coronary artery, with the subsequent risk of dissection for no medical indication. This pertains to patients without CAD and patients with CAD who did not undergo PCI, FFR, or IVUS. In total, coronary blood samples were collected in 23 out of 48 CAD patients.

In NSTEMI patients, coronary blood was sampled in eight of the 15 patients. In the remaining seven patients, the stenosis was considered too complex or too distal by the interventional cardiologist to allow for coronary blood extraction.

Prior to the inclusion of the first patient, we tested the use of different catheters for blood extraction to assure that it had no influence on plasma eCB levels. For this purpose, blood was drawn into a syringe and subsequently into additional syringes via either a Terumo 6F sheath, a pigtail catheter, or a 1.5 mm OTW balloon dilatation catheter. The passage of blood through the different catheters did not lead to artificial alterations of eCB levels (S1 Fig).

Additional sites for blood extraction in patients undergoing combined right- and left-heart catherterisation were the femoral vein, the inferior vena cava, the superior vena cava, the right ventricle, and the pulmonary artery. Blood from the femoral vein was drawn from the side port of the venous sheath (Terumo 6F; Terumo), whereas blood from each of the other sites within the low-pressure system was drawn using a pulmonary wedge pressure catheter (Medtronic, Minneapolis, USA).

After the sample was collected, the blood was immediately transferred to wet ice in order to limit *ex vivo* synthesis or degradation of plasma eCB. Thereafter, blood samples were transferred into a precooled Eppendorf 5810R centrifuge (Eppendorf, Hamburg, Germany) where they were spun at 2,000xg and 4°C for 10 minutes. Following centrifugation, the upper plasma phase was carefully transferred into precooled (4°C) 1.5 ml Eppendorf tubes and snap frozen in liquid nitrogen before being stored at -80°C until the LC/MRM analyses were performed.

### 2.3 Quantification of plasma endocannabinoid concentrations by LC/MRM

Concentrations of 2-AG, AEA, PEA, and AA were assessed by LC/MRM as previously described [21]. Briefly, 50 μl of a stock solution of internal standards and 250 μl ethyl acetate/n-hexane (9:1, v/v) were added to 100 μl of plasma. The samples were vortexed, centrifuged (10 minutes; 16,000xg; 4°C), and subsequently kept at -20°C for 10 min. The upper organic phase was transferred to 96-well plates, evaporated to dryness, and the extract was reconstituted in 50 μl acetonitrile/H_2_O (1:1, v/v) for LC/MRM analysis. Plasma sampling and eCB extraction methods follow highly standardised procedures, in order to minimise artificial alteration of the endogenous levels of the eCB. Blood sampling methods with subsequent eCB extraction were validated by measuring multiple samples from identical patients and identical locations, which showed low intra-individual variances.

### 2.4 Statistical analyses

Data are presented as the mean ± SEM for continuous and normally distributed variables. The prevalence of categorical variables is presented as absolute numbers and the respective proportion as a percentage in square brackets. Statistical analysis was performed by using GraphPad Prism (GraphPad Software, San Diego, USA). For the comparison of two groups, an unpaired Student’s two-sided t-test was applied. For the comparison of three or more groups, a one-way ANOVA and subsequent Bonferroni correction was performed. Distribution of cardiovascular risk factors and the use of medication among patient groups were analysed using Fisher’s exact test for retrospective data. Correlations between two continuous variables were calculated as Pearson’s correlation coefficients (r). P-values < 0.05 were considered statistically significant.

## Results

Endocannabinoids modulate inflammation via autocrine and paracrine mechanisms. Given that their synthesis and release is localised at sites of inflammation as well as their short half-life, we suspected that eCB levels vary locally and peak at sites of vascular inflammation.

### 3.1 Baseline characteristics

The present study includes 83 patients who received a coronary angiogram for suspected coronary artery disease. Patients were divided into three groups based on their final diagnosis: no-CAD (n = 20), CAD (n = 48), or NSTEMI (n = 15). Patient characteristics are listed in Table 1 and laboratory findings are detailed in Table 2. Except for hypercholesterolemia, which was more common in CAD patients than in no-CAD patients, cardiovascular risk factors were evenly distributed between the three groups. Statin use was higher in CAD patients compared to the two other groups, paralleled by lower total levels of cholesterol and low-density lipoprotein (LDL) in CAD patients as compared to no-CAD and NSTEMI patients. The use of aspirin and P2Y_12_ inhibitors on admission was more frequent in CAD- and NSTEMI patients compared to patients without coronary artery disease and prescription of ACE inhibitors and β-blockers was more common among CAD- than among no-CAD patients.

**Table 1:** Patient characteristics.

Cardiovascular risk factors	No CAD	CAD	NSTEMI
Age	61.5 (53–78)	68.5 (63–74)	67.0 (52–80)
Male sex	14 [70.0%]	44 [91.7%]	14 [93.3%]
Smoking			
current	2 [11.8%]	17 [35.4%]	6 [40.0%]
former	5 [29.4%]	13 [27.1%]	2 [13.3%]
never [1-x]	10 [58.8%]	18 [37.5%]	7 [46.7%]
Arterial hypertension	14 [77.8%]	39 [81.3%]	10 [66.7%]
Diabetes mellitus	5 [29.4%]	16 [33.3%]	2 [13.3%]
Hypercholesterolemia	10 [50%]	39 [81.3%]*	10 [66.7%]
Hyperlipidemia	5 [27.8%]	21 [43.8%]	4 [28.6%]
Family History	5 [27.8%]	7 [14.6%]	1 [6.7%]
Obesity BMI > 25 kg/m^2^	10 [52.6%]	36 [75.0%]	9 [60.0%]
**Co-morbidities**			
PAD	0 [0.0%]	12 [25.0%]*	0 [0.0%]
Previous myocardial infarction	0 [0.0%]	27 [56.3%]***	1 [6.7%]^§§§^
Stroke	3 [15.0%]	4 [8.3%]	1 [6.7%]
TIA	1 [5.0%]	1 [2.1%]	1 [6.7%]
Systolic heart failure	5 [25.0%]	18 [37.5%]	7 [46.7%]
Cardiomyopathy	2 [10.0%]	3 [6.25%]	1 [6.7%]
Valvulopathy	7 [35.0%]	22 [45.8%]	9 [60.0%]
DVT	1 [5.0%]	3 [6.25%]	0 [0.0%]
Present or former PCI	0 [0.0%]	40 [83.3%]***	14 [93.3%]***
Liver dysfunction	0 [0.0%]	2 [4.2%]	0 [0.0%]
**Medication on admission**			
Oral anticoagulation	5 [25.0%]	16 [33.3%]	4 [26.7%]
Aspirin	7 [35.0%]	32 [69.6%]*	14 [93.3%]***
P2Y_12_ inhibitors	1 [5.0%]	23 [50.0%]***	9 [60.0%]***
β-blockers	8 [40.0%]	35 [76.1%]*	10 [66.7%]
ACE inhibitors	7 [35.0%]	33 [68.8%]*	6 [40.0%]
AT1 receptor antagonists	5 [25.0%]	9 [18.8%]	2 [13.3%]
Diuretics	8 [40.0%]	20 [42.6%]	5 [33.3%]
Calcium antagonists	3 [15.0%]	8 [16.7%]	2 [13.3%]
Antiarrhythmics	3 [15.0%]	8 [16.7%]	1 [6.7%]
Statins	5 [25.0%]	36 [78.3%]***	9 [60.0%]
PPI	9 [45.0%]	24 [51.1%]	7 [46.7%]
**Access**			
Radial access	18 [90.0%]	34 [70.8%]	13 [86.7%]
Femoral access	2 [10.0%]	14 [29.2%]	2 [13.3%]

Data are presented as the median (95% CI) for continuous and normally distributed variables. The prevalence of categorical variables is presented as absolute numbers and the respective proportion as a percentage in square brackets.

* p_vs. no-CAD_ < 0.05,

*** p_vs. no-CAD_ < 0.001,

^§§§^ p_vs. CAD_ < 0.001, assessed by ANOVA with subsequent Bonferroni correction for continuous and normally distributed variables and by Fisher’s exact test for retrospective data for categorical variables. A family history of myocardial infarction was considered as a cardiovascular risk factor when the event occurred in a female first-degree relative before the age of 65 or in a male first-degree relative before the age of 55.

AT1, angiotensin II type 1; BMI, body mass index; CAD, coronary artery disease; DVT, deep vein thrombosis; LAD, left anterior descending artery; LCx, left circumflex artery; NSTEMI, non-ST-segment elevation myocardial infarction; PAD, peripheral artery disease; PPI, proton pump inhibitor; RCA, right coronary artery; TIA, transient ischemic attack.

**Table 2:** Laboratory findings.

	No CAD	CAD	NSTEMI
Erythrocytes [T/L]	4.8 (4.6–5.0)	4.6 (4.2–4.7)	4.4 (3.6–4.9)
Hb [g/dl]	14.0 (13.8–14.8)	13.4 (12.7–14.3)	13.6 (11.3–15.3)
Hematocrit [%]	42.0 (40.0–44.0)	39.5 (38.0–41.0)	39.0 (34.0–44.0)
Thrombocytes [g/l]	218 (161–252)	232 (196–256)	245 (185–258)
Leukocytes [g/l]	7.9 (6.1–8.6)	8.1 (6.9–8.7)	7.3 (6.8–9.8)
Lymphocytes [g/l]	2.0 (1.6–2.6)	1.9 (1.7–2.1)	2.0 (1.1–3.7)
Monocytes [g/l]	0.7 (0.6–0.8)	0.6 (0.6–0.7)	0.8 (0.6–0.9)
Neutrophil Granulocytes [g/l]	4.6 (3.6–6.1)	4.8 (4.1–5.5)	4.7 (3.5–5.1)
Eosinophil Granulocytes [g/l]	0.1 (0.1–0.2)	0.2 (0.1–0.2)	0.2 (0.2–0.3)
Basophil Granulocytes [g/l]	0.05 (0.03–0.07)	0.05 (0.04–0.06)	0.04 (0.03–0.07)
CRP [mg/l]	2.7 (1.1–6.7)	2.6 (1.5–4.7)	9.4 (3.0–28.8)
PCT [μg/l]	0.08 (0.04–0.09)	0.06 (0.05–0.07)	0.07 (0.04–0.09)
IL-6 [pg/ml]	2.6 (2.0–25.1)	3.0 (2.0–4.8)	7.8 (3.4–16.4)
IL-8 [pg/ml]	11.6 (7.4–28.6)	10.7 (9.1–13.1)	7.6 (5.8–14.9)
Complement 3 [g/L]	1.2 (1.1–1.4)	1.2 (1.1–1.4)	1.2 (1.0–1.4)
Complement 4 [g/L]	0.3 (0.2–0.3)	0.3 (0.3–0.3)	0.3 (0.2–0.3)
HbA1c [%]	5.9 (5.4–6.6)	5.9 (5.7–6.3)	5.8 (5.5–6.5)
Peak troponin [ng/ml]	0.0 (0.0–0.3)	0.0 (0.0–0.0)	2.5 (0.3–6.8)*^,^^§§^
Peak CK-MB [μg/l]	0.0 (0.0–1.3)	0.0 (0.0–0.0)	1.7 (0.5–4.4)^§^
Total cholesterol [mg/dl]	200 (155–233)	155 (144–172)*	191 (129–261)
LDL [mg/dl]	120 (93–129)	92 (78–101)*	109 (68–144)
HDL [mg/dl]	53 (39–59)	46 (39–54)	48 (37–53)
Triglycerides [mg/dl]	103 (70–179)	131 (107–161)	127 (108–181)

Data are presented as the median (95% CI) for continuous and normally distributed variables. P-values:

* p_vs. no-CAD_ < 0.05,

^§^ p_vs. CAD_ < 0.05,

^§§^ p_vs. CAD_ < 0.01, assessed by ANOVA with subsequent Bonferroni correction.

CK-MB, creatine kinase-muscle/brain; CRP, c-reactive protein; Hb, hemoglobin; HDL, high-density lipoprotein; IL, interleukin; LDL, low-density lipoprotein; PCT, procalcitonin.

### 3.2 Arterial 2-AG levels vary both regionally and between patient groups

Arterial eCB levels were measured in the sheath, aorta, and in the coronary artery distal to the culprit lesion. Plasma concentrations of 2-AG, AEA, PEA, and AA were quantified using LC/MRM.

**Fig 1:**
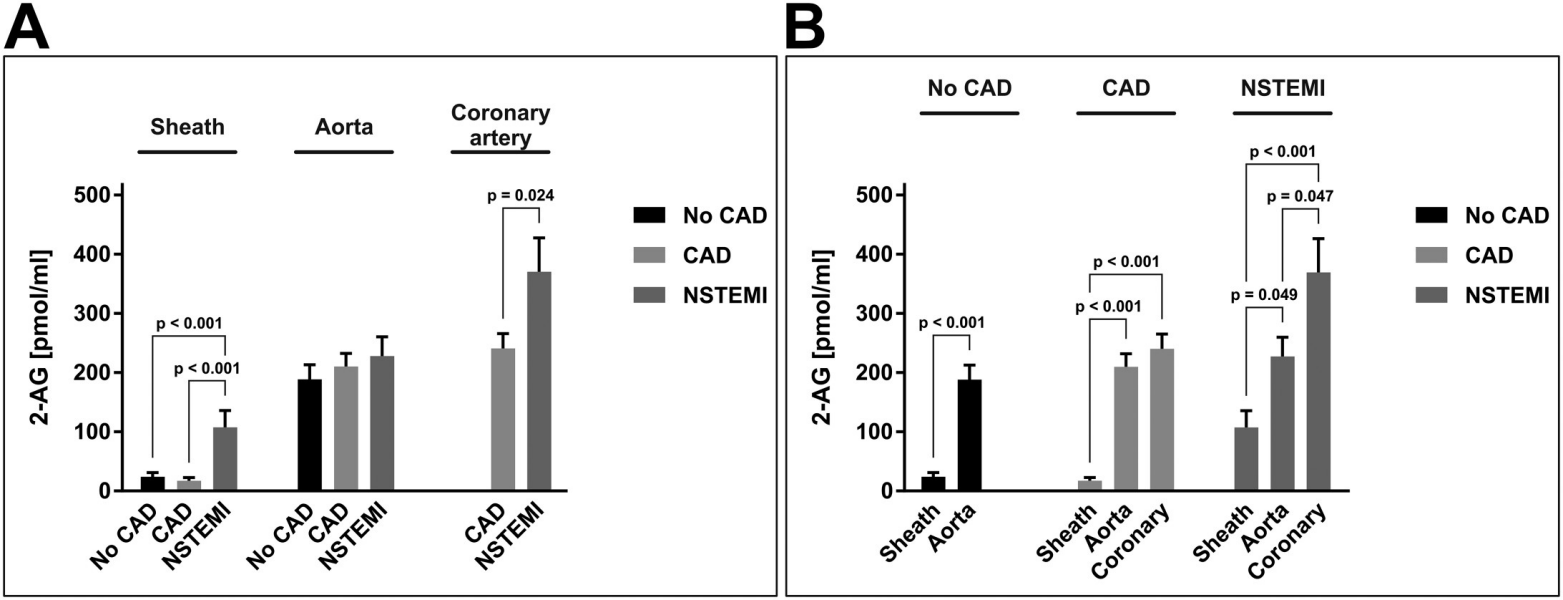
Arterial concentrations of 2-arachidonoylglycerol. Panels A and B depict the same data of arterial concentrations of 2-AG [pmol/ml]. Data are presented as the mean ± standard error of the mean. For the purpose of clarity, values in panel (A) are grouped by extraction site, allowing for comparison of different patient groups at one site. Values in panel (B) are grouped by patient group affiliation, allowing for comparison of different blood extraction sites within one patient group. N = 83 patients. P-values as indicated, assessed by ANOVA with subsequent Bonferroni correction. 2-AG, 2-arachidonoylglycerol; CAD, coronary artery disease; coronary, culprit coronary artery; NSTEMI, non-ST-segment elevation myocardial infarction.

Interestingly, 2-AG showed a marked variability both between patient groups and the sites of blood extraction. At all three blood extraction sites, NSTEMI patients consistently yielded the highest plasma levels of 2-AG, as compared to no-CAD and CAD patients. These differences were most pronounced in samples drawn from the sheath, where NSTEMI patients showed a 2-AG level of 107.4 ± 28.4 pmol/ml, whereas no-CAD and CAD patients yielded 23.9 ± 7.1 pmol/ml (p_vs. NSTEMI_ < 0.001) and 17.4 ± 5.4 pmol/ml (p_vs. NSTEMI_ < 0.001) 2-AG, respectively (Fig 1A).

In aortic blood samples, the differences between patient groups were less pronounced, and only a non-significant tendency towards higher 2-AG levels in samples from NSTEMI patients was detected (227.2 ± 32.6 pmol/ml (NSTEMI) vs. 188.2 ± 24.5 pmol/ml (no-CAD; p_vs. NSTEMI_ > 0.05) and 209.7 ± 22.1 pmol/ml (CAD; p_vs. NSTEMI_ > 0.05; Fig 1A). Surprisingly, 2-AG levels were consistently higher in aortic blood samples than in blood samples drawn from the sheath, irrespective of classification to a certain patient group (no-CAD: 188.2 ± 24.5 pmol/ml vs. 23.9 ± 7.1 pmol/ml (p_vs. Aorta_ < 0.001); CAD: 209.7 ± 22.1 pmol/ml vs. 17.4 ± 5.4pmol/ml (p_vs. Aorta_ < 0.001); NSTEMI: 227.2 ± 32.6 pmol/ml vs. 107.4 ± 28.4 pmol/ml (p_vs. Aorta_ = 0.049; Fig 1B)).

The highest concentration of 2-AG was detected in coronary blood from NSTEMI patients (369.3 ± 57.2 pmol/ml), which was significantly higher than in coronary blood from CAD patients (240.1 ± 25.3 pmol/ml; p_vs. NSTEMI_ = 0.024; Fig 1A). In NSTEMI patients, the concentration of 2-AG in the coronary arteries was significantly higher than in the respective aortic blood samples (369.3 ± 57.2 pmol/ml vs. 227.2 ± 32.6 pmol/ml; p = 0.047; Fig 1B). This was not the case in CAD patients, for whom 2-AG levels in the coronary arteries were only slightly higher than in the respective aortic blood samples, without reaching statistical significance (240.1 ± 25.3 pmol/ml vs. 209.7 ± 22.1 pmol/ml; p > 0.05; Fig 1B).

### 3.3 *N*-arachidonoylethanolamide, palmitoylethanolamide, and arachidonic acid levels do not exhibit pronounced differences between blood extraction sites or patient groups

While 2-AG levels varied considerably between patient groups and blood extraction sites, only a minor variability was observed for the endocannabinoid AEA, for the endocannabinoid-related compound PEA and for arachidonic acid.

**Fig 2:**
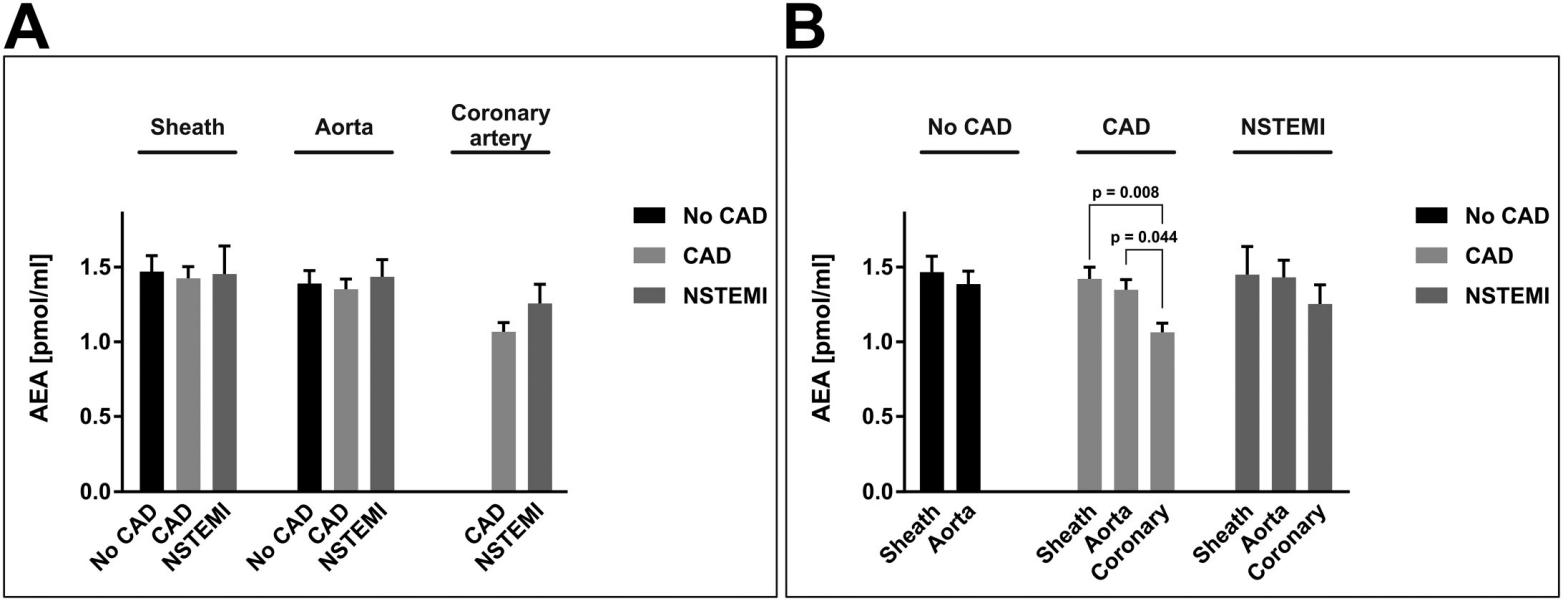
Arterial concentrations of *N*-arachidonoylethanolamide. Panels A and B depict the same data of arterial concentrations of AEA [pmol/ml]. Data are presented as the mean ± standard error of the mean. For the purpose of clarity, values in panel (A) are grouped by extraction site, allowing for comparison of different patient groups at one site. Values in panel (B) are grouped by patient group affiliation, allowing for comparison of different blood extraction sites within one patient group. N = 83 patients. P-values as indicated, assessed by ANOVA with subsequent Bonferroni correction. AEA, *N*-arachidonoylethanolamide; CAD, coronary artery disease; coronary, culprit coronary artery; NSTEMI, non-ST-segment elevation myocardial infarction.

As reported for AEA plasma levels from venous blood, arterial plasma concentrations of AEA were approximately 10 to 100-fold lower than the respective 2-AG levels. In our measurements, AEA levels showed only slight differences between extraction sites and none between patient groups. In CAD patients, coronary AEA levels were slightly lower than AEA levels in the sheath (1.1 ± 0.1 pmol/ml vs. 1.4 ± 0.1 pmol/ml; p = 0.008) and the aorta 1.4 ± 0.1 pmol/ml; p_vs. coronary artery_ = 0.044), while in the other two patient groups, there were no regional differences between the three extraction sites. AEA levels were 1.5 ± 0.1 pmol/ml and 1.4 ± 0.1 pmol/ml (sheath and aorta) in no-CAD patients, and 1.4 ± 0.1 pmol/ml, 1.4 ± 0.1 pmol/ml, and 1.1 ± 0.1 pmol/ml (sheath, aorta, and coronary artery) in NSTEMI patients (Fig 2).

**Fig 3:**
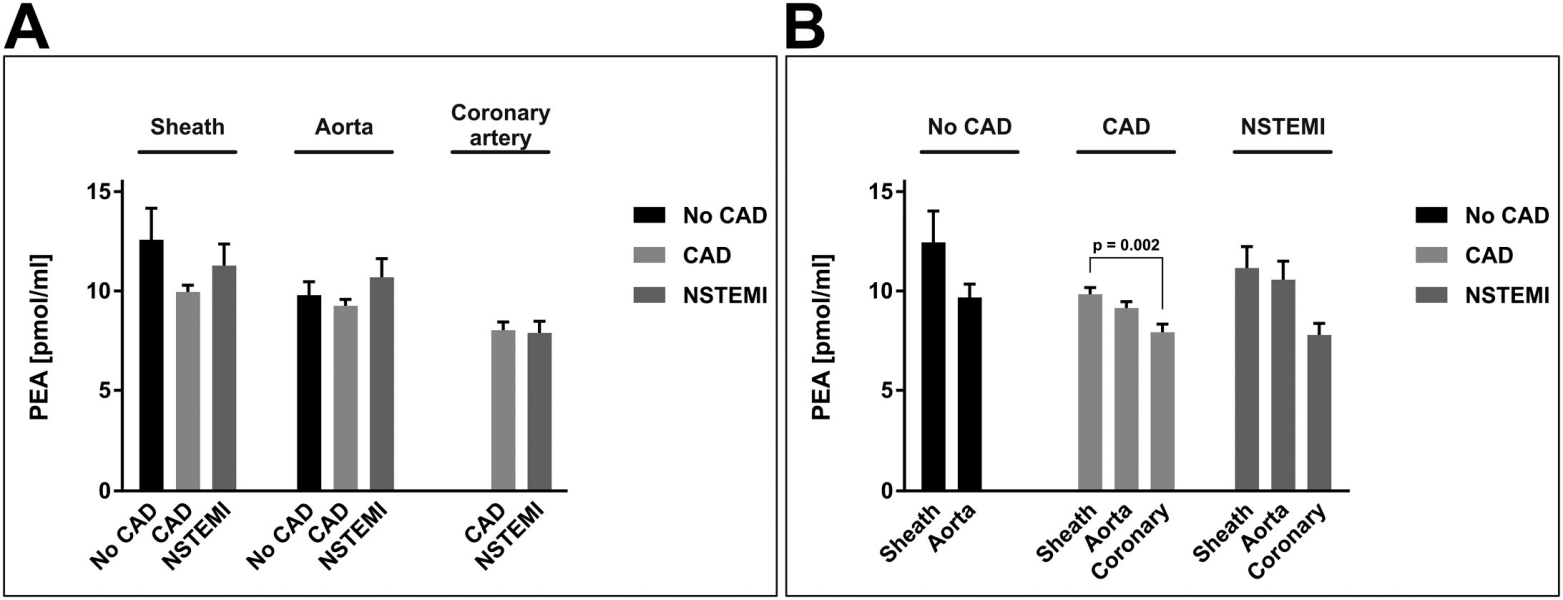
Arterial concentrations of palmitoylethanolamide. Panels A and B depict the same data of arterial concentrations of PEA [pmol/ml]. Data are presented as the mean ± standard error of the mean. For the purpose of clarity, values in panel (A) are grouped by extraction site, allowing for comparison of different patient groups at one site. Values in panel (B) are grouped by patient group affiliation, allowing for comparison of different blood extraction sites within one patient group. N = 83 patients. P-values as indicated, assessed by ANOVA with subsequent Bonferroni correction. CAD, coronary artery disease; coronary, culprit coronary artery; NSTEMI, non-ST-segment elevation myocardial infarction; PEA, palmitoylethanolamide.

For PEA, we detected only subtle differences between the sites of blood extraction, while there were no statistically significant differences between patient groups. In all three patient groups, PEA concentrations were slightly lower in coronary and aortic blood as compared to blood from the arterial sheath. This reached statistical significance in the CAD patient group, where PEA levels in coronary blood were 8.0 ± 0.4 pmol/ml compared to 10.0 ± 0.3 pmol/ml in the sheath (p = 0.002; Fig 3).

**Fig 4:**
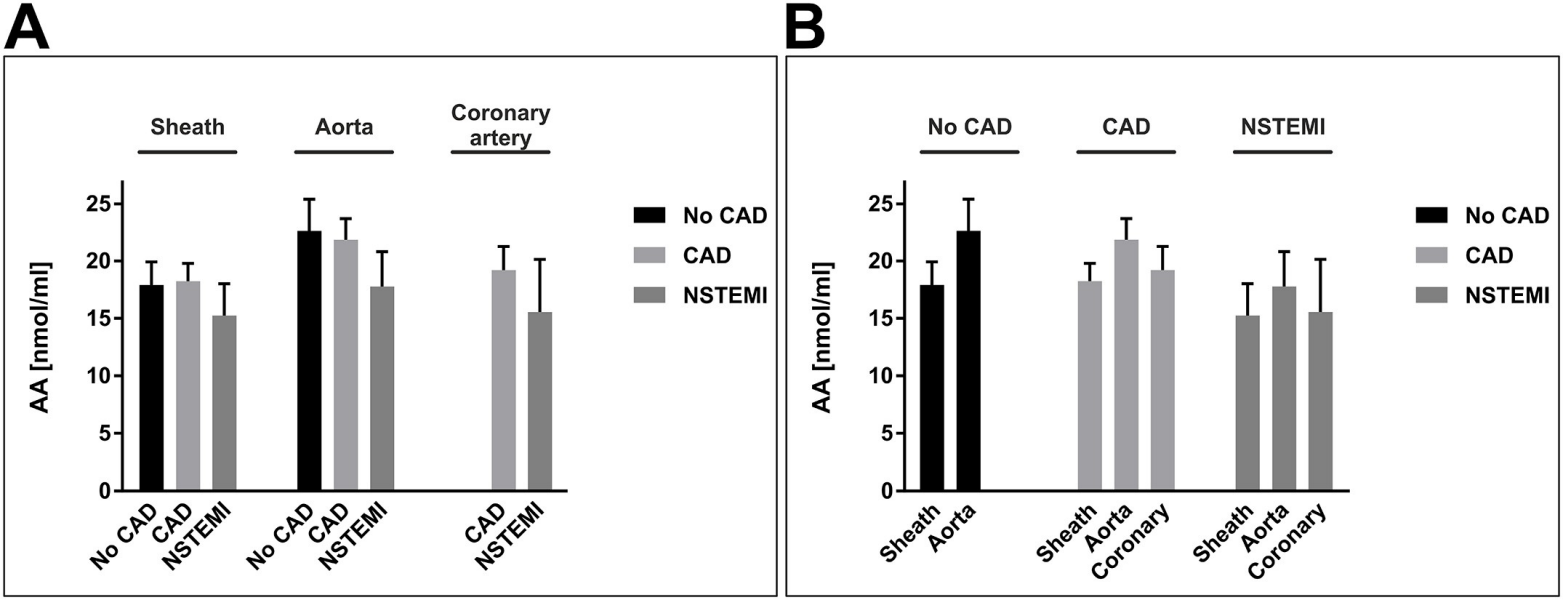
Arterial concentrations of arachidonic acid. Panels A and B depict the same data of arterial concentrations of AA [nmol/ml]. Data are presented as the mean ± standard error of the mean. For the purpose of clarity, values in panel (A) are grouped by extraction site, allowing for comparison of different patient groups at one site. Values in panel (B) are grouped by patient group affiliation, allowing for comparison of different blood extraction sites within one patient group. N = 83 patients; p > 0.05, assessed by ANOVA with subsequent Bonferroni correction. AA, arachidonic acid; CAD, coronary artery disease; coronary, culprit coronary artery; NSTEMI, non-ST-segment elevation myocardial infarction.

Finally, we analysed plasma levels of arachidonic acid, which is both, a precursor lipid and a degradation product of AEA and 2-AG, and found no statistically significant differences between patient groups or extraction sites (Fig 4). AA levels yielded 17.9 ± 2.0 nmol/ml and 22.6 ± 2.8 nmol/ml (sheath, aorta) in no-CAD patients, 18.2 ± 1.6 nmol/ml, 21.8 ± 1.8 nmol/ml, and 19.2 ± 2.0 nmol/ml (sheath, aorta, and coronary artery) in CAD patients, and 15.2 ± 2.8 nmol/ml, 17.8 ± 3.0 nmol/ml, and 15.6 ± 4.6 nmol/ml (sheath, aorta, and coronary artery) in NSTEMI patients.

### 3.4 2-AG levels neither differ between radial and femoral access nor between LAD, LCx, and RCA

We subsequently analysed, whether the location of the sheath (radial vs. femoral access) influenced arterial 2-AG concentrations. The no-CAD and NSTEMI patient groups only comprised 2 patients each in whom coronary angiography was performed via a femoral access [10.0% and 13.3%] and, thus, did not allow statistical analyses. In the CAD group, radial access was obtained in 34 out of 48 patients [70.8%] and femoral access was chosen in 14 patients [29.2%]. 2-AG levels showed no differences between radial and femoral access and averaged 17.8 ± 7.1 pmol/ml in radial sheaths vs. 16.4 ± 7.0 pmol/ml in femoral sheaths (p > 0.05).

Similarly, plasma 2-AG levels did not differ between the diseased coronary arteries in the CAD group. Coronary blood was drawn from the vessel that underwent PCI, which was the left anterior descending artery (LAD) in 9 cases, left circumflex artery (LCx) in 5, and the right coronary artery (RCA) in 8 cases (1 intermediate artery was not included in this analysis). 2-AG levels did not reach statistically significant differences between the three vessels, yielding 262.7 ± 50.1 pmol/ml vs. 280.7 ± 61.9 pmol/ml, and 195.9 ± 25.0 pmol/ml in LAD, LCx, and RCA (p > 0.05).

### 3.5 2-AG levels do not peak within the low-pressure system

**Fig 5:**
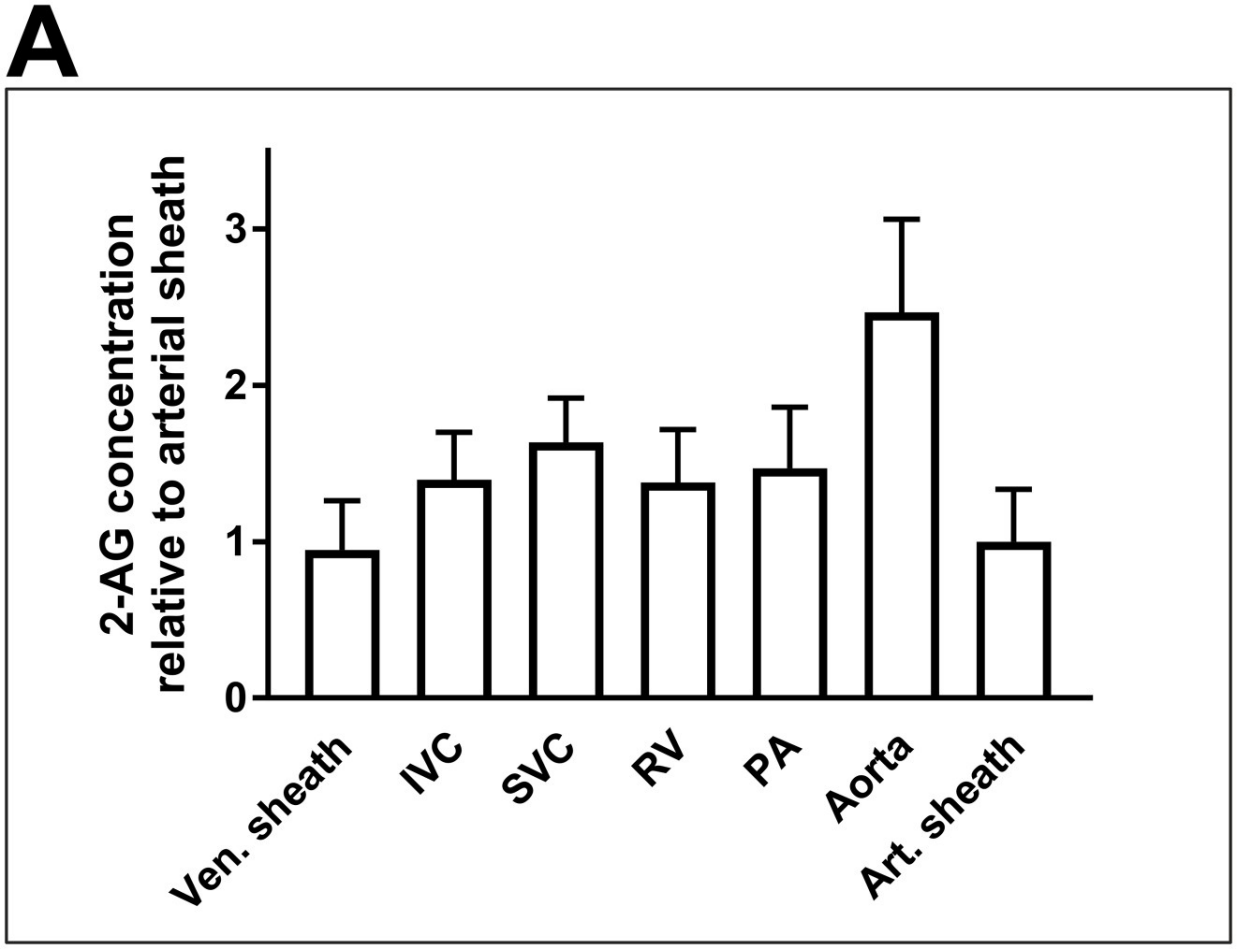
Concentrations of 2-AG do not peak within the low-pressure system. Blood samples were drawn at distinct locations during combined right- and left-heart catheterisation. Sites of blood extraction were the venous sheath, the inferior vena cava (IVC), the superior vena cava (SVC), the right ventricle (RV), and the pulmonary artery (PA), as well as the aorta and the arterial sheath. Data are presented as the mean ± standard error of the mean. N = 5 patients; p > 0.05, assessed by ANOVA with subsequent Bonferroni correction. 2-AG, 2-arachidonoylglycerol.

Because 2-AG levels appeared to peak in the aorta and then sharply decreased towards the peripheral arteries, we sought to further localise the site of 2-AG synthesis. In an attempt to determine whether 2-AG is synthesised before or after the pulmonary capillary bed, we measured 2-AG levels in patients, who underwent combined right- and left-heart catheterisation. However, there was no abrupt leap in 2-AG concentrations within the low-pressure system, which could have been indicative of local 2-AG synthesis. Instead, 2-AG levels yielded 26.5 ± 8.9 pmol/ml in the femoral vein and 39.1 ± 8.5 pmol/ml and 45.8 ± 7.9 pmol/ml respectively in the inferior and superior vena cava. In the right ventricle and pulmonary artery, 2-AG concentrations reached 38.6 ± 9.5 pmol/ml and 41.1 ± 11.0 pmol/ml, respectively. None of these 2-AG concentrations were statistically different one from another (Fig 5).

Similarly, AEA, PEA and AA levels remained entirely unaltered throughout the low-pressure system. The respective concentrations are detailed in S2 Fig.

### 3.6 Correlation of endocannabinoid levels with clinical parameters and laboratory findings

eCB levels were correlated with clinical parameters and laboratory findings. We suspected eCB concentrations in the blood sample drawn from the sheath to depend the least on local synthesis and to be most representative of systemic eCB levels. These values were correlated with clinical parameters and laboratory values that were obtained from a venous blood sample that had been drawn upon admission to the hospital. Laboratory values comprised a complete blood count, inflammatory parameters, cardiac troponin, creatine kinase-muscle/brain (CK-MB), Hba1c, and a lipid profile. 2-AG levels correlated with peak troponin (r = 0.46; p < 0.001) and with IL-6 (r = 0.42; p = 0.001). Pearson’s correlation coefficients of 2-AG, AEA, PEA, and AA with clinical parameters and laboratory findings are detailed in Table 3. A version of this table containing all r- and p-values is available in the supporting information (S1 Table).

**Table 3:** Pearson’s correlation coefficients (r) of endocannabinoid levels (sheath) with clinical parameters and laboratory findings.

Correlation with	2-AG	AEA	PEA	AA
Age	ns	ns	ns	ns
Weight	ns	ns	ns	ns
Height	ns	ns	ns	ns
BMI	ns	r = 0.3703 p = 0.0069	ns	r = 0.3215 p = 0.0259
Systolic blood pressure	ns	ns	ns	ns
Diastolic blood pressure	ns	r = 0.2502 p = 0.0315	ns	ns
Heart rate	ns	r = 0.2553 p = 0.0293	r = 0.3478 p = 0.0042	ns
Erythrocytes	ns	r = 0.3045 p = 0.0083	ns	r = 0.2524 p = 0.0379
Hb	ns	r = 0.2765 p = 0.0171	r = 0.2660 p = 0.0296	r = 0.2596 p = 0.0325
Hematocrit	ns	r = 0.3086 p = 0.0075	r = 0.2867 p = 0.0186	ns
Thrombocytes	ns	ns	ns	ns
Leukocytes	ns	ns	ns	ns
Lymphocytes	ns	ns	ns	ns
Monocytes	ns	ns	ns	ns
Neutrophil Granulocytes	ns	ns	ns	ns
Eosinophil Granulocytes	ns	ns	ns	ns
Basophil Granulocytes	ns	ns	ns	ns
CRP	ns	ns	ns	ns
PCT	ns	ns	ns	ns
IL-6	r = 0.4235 p = 0.0013	ns	ns	ns
IL-8	ns	ns	ns	ns
Complement 3	ns	ns	ns	ns
Complement 4	ns	ns	ns	ns
HbA1c	ns	ns	ns	ns
Peak troponin	r = 0.4564 p < 0.0001	ns	ns	ns
Peak CK-MB	ns	ns	ns	ns
Total cholesterol	ns	ns	r = 0.2556 p = 0.0468	ns
LDL	ns	ns	r = 0.2990 p = 0.0147	ns
HDL	ns	ns	ns	ns
Triglycerides	ns	ns	ns	ns

P-values as indicated. 2-AG, 2-arachidonoylglycerol; AA, arachidonic acid; AEA, *N*-arachidonoylethanolamide; BMI, body mass index; CK-MB, creatine kinase-muscle/brain; CRP, c-reactive protein; Hb, hemoglobin; HDL, high-density lipoprotein; IL, interleukin; LDL, low-density lipoproetin; PCT, procalcitonin; PEA, palmitoylethanolamide.

### 3.7 Intake of antiplatelet and antidyslipidemic agents does not significantly influence 2-AG levels

Influence of pharmacological therapy with antiplatelet and antidyslipidemic agents on 2-AG levels was assessed. Subgroups were formed from the CAD group based on whether or not patients were prescribed aspirin, P2Y12 inhibitors, or statins at the time of blood sampling. 2-AG concentrations in blood samples drawn from the sheath were used for statistical analyses.

Aspirin: In the CAD group, 32 out of 48 patients were on aspirin at the time of inclusion, 14 patients of this group were not on aspirin. Information on aspirin prescription is missing for 2 patients of this group. 2-AG levels among patients taking aspirin were not significantly different from those not taking aspirin (20.2 ± 7.7 pmol/ml vs. 11.4 ± 3.9 pmol/ml; p = 0.4543).

P2Y12 inhibitors: In the CAD group, 23 out of 48 patients were on a P2Y12 inhibitor at the time of inclusion, 23 patients of this group were not on a P2Y12 inhibitor. Information on P2Y12 inhibitor prescription is missing for 2 patients of this group. Again, 2-AG levels did not differ between the two subgroups yielding 21.8 ± 9.9 pmol/ml among patients taking a P2Y12 inhibitor compared to 12.9 ± 4.2 pmol/ml in patients not taking a P2Y12 inhibitor (p = 0.5245).

Statins: In the CAD group, 36 out of 48 patients were on a statin at the time of inclusion, 10 patients of this group were not on a statin. Information on statin prescription is missing for 2 patients of this group. As for antiplatelet drugs, intake of statins did not significantly modify 2-AG levels. These yielded 16.0 ± 6.0 pmol/ml among patients on a statin compared to 27.1 ± 19.5 pmol/ml in patients not taking a statin (p = 0.4200).

## Discussion

Nearly a decade ago, Sugamura and coworkers demonstrated eCB levels to be significantly higher in patients with coronary artery disease than in patients without. This observation was based on eCB levels measured in venous blood samples from CAD- and no-CAD patients [17]. However, eCB have a short half-life and act via autocrine and paracrine pathways, therefore eCB levels in venous blood samples might neither reflect coronary eCB concentrations nor determine eCB biology within atherosclerotic coronary arteries. Arguably, changes of the eCB levels in venous blood from CAD patients, as observed by Sugamura, might stem from other metabolic consequences of the CAD pathology and do not unequivocally demonstrate local effects of eCB at the coronary arteries.

In the present study, we aimed at overcoming the limitations that arise from venous blood sampling. We therefore sampled arterial blood at distinct locations (sheath, aorta, culprit coronary artery) and analysed plasma endocannabinoid concentrations by using LC/MRM.

Our data show an unexpectedly marked variability in 2-AG concentrations between the sites of blood extraction and between patient groups, whereas AEA concentrations were evenly distributed in all samples. High 2-AG concentrations within the aortic root and the coronary arteries indicate that this endocannabinoid is likely synthesised at these locations. Coronary arteries of NSTEMI patients showed the highest 2-AG levels, being significantly higher than 2-AG levels in coronary arteries of CAD patients. Lesional macrophages might be responsible for the increase in 2-AG concentrations within the coronary arteries of NSTEMI patients, given their abundance in inflammatory plaques. However, other circulating and resident vascular cell types have also been described to produce 2-AG and might account for an increase in 2-AG during myocardial infarction: first, platelets have been described to release 2-AG upon treatment with platelet-activating factor (PAF) [22]. Interestingly, treatment with PAF does not increase AEA secretion [22]. Synthesis of 2-AG but not AEA by activated thrombocytes could explain why 2-AG concentrations vary locally, whereas AEA concentrations do not. Second, T- and B-cells have been demonstrated to release 2-AG upon activation [23]. Yet, given their small number in atherosclerotic plaques, their overall contribution to local blood endocannabinoid levels seems unlikely. While all of these cells may contribute to blood eCB concentrations, there is currently no study demonstrating which blood cells mainly synthesise eCB under physiological and pathophysiological conditions.

NSTEMI patients appeared not only to display increased 2-AG levels within their coronary arteries, but also showed a significant increase of 2-AG in peripheral arteries compared to CAD- and no-CAD patients. It might be speculated that this arises from the systemic inflammatory response that accompanies myocardial infarction (reviewed by [24]). Interestingly, there was no significant difference in peripheral arterial 2-AG levels between CAD- and no-CAD patients. This seems at odds with findings by Sugamura et al., who reported an increase in venous concentrations of both AEA and 2-AG in CAD over no-CAD patients [17]. AEA, however, was not elevated in any of the patient groups in the present study nor was it elevated at any particular site. This finding is in line with the study by Maeda and coworkers, who reported AEA to be elevated in the coronary arteries of STEMI patients, but not in those of patients with stable angina [18]. This difference between STEMI and NSTEMI patients might represent different molecular mechanisms that emphasise the distinct characteristics of the two entities, STEMI and NSTEMI.

Interestingly, both AA and AEA showed a significant positive correlation with BMI, while 2-AG did not correlate with BMI in our study. In 2012, Quercioli and colleagues also described a positive correlation between BMI and the endocannabinoid AEA (r = 0.35; p = 0.0001) in a cohort of 111 individuals divided into 4 groups based on their BMI with an interquartile range of 21.9 to 45.0 kg/m^2^ in the entire study population [7]. Meanwhile there was no significant correlation between BMI and 2-AG in this study (r = 0.14; p = 0.167) [7]. Our own data corroborate the findings by Quercioli et al.. Based these data, one might speculate that relevant amounts of AEA and AA are synthesised from adipose tissue, whereas 2-AG is not.

Elevated 2-AG levels in the culprit coronary artery of NSTEMI patients might be of pathophysiological relevance to the course of myocardial infarction, because 2-AG could promote thrombus formation. 2-AG has been shown to cause platelet activation and shape change at high concentrations of about 100 μM [25]. Although these high concentrations are well above what we detected in the present study (400 nM), 2-AG might facilitate platelet aggregation at lower concentrations in the thrombogenic setting of a myocardial infarction. Interestingly, our study found a correlation between 2-AG levels and IL-6, the latter of which has recently been demonstrated to be associated with an elevated risk of major adverse cardiovascular events after an acute coronary syndrome [26]. Furthermore, IL-6 appears to be crucial to human atherogenesis in two contemporary anti-inflammatory trials [27, 28]. Thus, the correlation of 2-AG with IL-6 might add to the clinical relevance of 2-AG in NSTEMI patients. While Maeda and coworkers found rather protective effects of elevated AEA levels following myocardial infarction in regard to left ventricular function, Quercioli et al. demonstrated a negative correlation of both, 2-AG and AEA, with coronary blood flow in obese patients [6, 7, 18]. Clearly, the impact of 2-AG levels on the clinical outcome of ACS patients needs to be determined in future studies.

One equally striking and unexpected finding of this study is the sharp decrease in 2-AG concentration that occurs from the aortic root to the peripheral arteries. This decrease is most pronounced in CAD- and no-CAD patients where peripheral 2-AG levels are as low as 20 pmol/ml compared to > 200 pmol/ml in the aorta. We have carefully ruled out the possibility that the passage of blood through a catheter alters plasma eCB concentrations—we found no artificial changes in eCB levels in control experiments. The observed decline in 2-AG concentration happens within seconds inside the vasculature and seems to depend on resident vascular cells, because aortic blood, when drawn using a catheter, naturally stays in contact with the surrounding circulating blood cells. 2-AG mainly breaks down via enzymatic degradation by monoacylglycerol lipase (MAGL). This degradation occurs quickly but not within seconds. Additionally, AA did not significantly change as a consequence of 2-AG degradation, pointing also towards an alternative cause for the observed decline in 2-AG concentrations. One mechanism that does work on a timescale of seconds is facilitated diffusion. It has long been proposed that putative transporters shuttle eCB inside cells, which rapidly decreases their concentration in the extracellular space (reviewed by [5]). If this mechanism applies here, one might hypothesise these transporters to be highly expressed in the endothelium of large human arteries.

Given that aortic 2-AG concentrations are consistently higher than peripheral 2-AG levels in all three patient groups, we sought to determine whether there is a locus for physiological 2-AG release prior to the heart but within the low-pressure system. We therefore analysed blood from patients undergoing combined left- and right-heart catheterisation. We drew blood from the venous sheath and from the inferior vena cava (IVC) as a reference. We also drew blood from the superior vena cava (SVC), because synthesis and release of 2-AG in the brain could contribute to blood endocannabinoid levels. In addition, we drew blood from the right ventricle, because nutritional lipids that are broken down in the liver could trigger 2-AG synthesis and a subsequent leap in 2-AG levels distal to the insertion of the hepatic veins might occur. Yet, there was no jump in 2-AG levels, neither in the SVC nor in the right ventricle, indicating that neither the CNS nor the liver contributes substantially to blood 2-AG levels. Naturally, 2-AG production from nutritional lipids may increase under non-fasting conditions, which we would not observe, because all patients had to be fasting prior to catheterisation. Finally, we drew blood from the pulmonary artery to check for localised synthesis of 2-AG, however, we found no evidence of this. In addition, pharmacological therapy with antiplatelet and antidyslipidemic agents may affect eCB synthesis and metabolism. Heterogeneity in drug prescription between the three patient groups might thus influence eCB levels. In subgroup analyses, we found no evidence of significant influence of antiplatelet and antidyslipidemic pharmacotherapy on 2-AG levels. Yet these cannot be ruled out, given the small size of these subgroups. However, differences in 2-AG levels at different sites of blood extraction within the same individual may not be readily explained by pharmacological influence. Likewise, one might suspect that pharmacological agents equally influence all endoannabinoids and eCB like compounds and not only 2-AG.

Our study has several limitations. Obviously, the small patient number poses a major limitation concerning the generalisability of our data. Given the small sample size, data might not be representative of the overall population of patients with NSTEMI or stable CAD. Second, the time that elapsed between the onset of symptoms and blood sampling was not uniform amongst NSTEMI patients. It is possible that 2-AG levels vary considerably over time within the same location, which is not represented adequately by a single measurement at a single time point. Finally, it would have been interesting to also measure eCB in STEMI patients; however, this was not possible due to ethical concerns regarding the patients’ ability to consent to our study in an emergency setting.

To our knowledge, this is the first study to systematically examine the local variability of eCB in no-CAD, CAD, and NSTEMI patients throughout the arterial and venous vasculature. In conclusion, our data show a marked variability of 2-AG, but not AEA, PEA, and AA, in arterial blood samples. This local variability could indicate local synthesis of 2-AG, which might in part reflect local vascular inflammation. Conceivably, inflammatory markers other than eCB may display similar characteristics. The clinical and prognostic relevance of elevated 2-AG levels in patients with acute coronary syndrome awaits clarification.

## Supporting information

S1 Fig Catheter-induced artificial alterations to eCB levels. eCB levels were not altered artificially by the method of blood extraction. In order to ensure that eCB concentrations are not artificially altered by the use of different sheaths and catheters, one identical blood sample was drawn through a sheath, a pigtail catheter, an over-the-wire (OTW) balloon dilatation catheter, and a pulmonary wedge pressure catheter which did not cause artificial alterations to the eCB levels. Data are presented as the mean ± standard error of the mean.
(TIF)

S2 Fig Concentrations of N-arachidonoylethanolamide, palmitoylethanolamide and arachidonic acid in the low-pressure system. Blood samples were drawn at distinct locations during combined right- and left-heart catheterization. Sites of blood extraction were the venous sheath, the inferior vena cava (IVC), the superior vena cava (SVC), the right ventricle (RV), and the pulmonary artery (PA), as well as the aorta and the arterial sheath. Data are presented as the mean ± standard error of the mean. AA, arachidonic acid; AEA, N-arachidonoylethanolamide; PEA, palmitoylethanolamide.
(TIF)

S1 Table Pearson’s correlation coefficients (r) of endocannabinoid levels (sheath) with clinical parameters and laboratory findings. P-values as indicated. 2-AG, 2-arachidonoylglycerol; AA, arachidonic acid; AEA, *N*-arachidonoylethanolamide; BMI, body mass index; CK-MB, creatine kinase-muscle/brain; CRP, c-reactive protein; Hb, hemoglobin; HDL, high-density lipoprotein; IL, interleukin; LDL, low-density lipoproetin; PCT, procalcitonin; PEA, palmitoylethanolamide.
(DOCX)

## Abbreviations

2-AG: 2-Arachidonoylglycerol
AA: Arachidonic acid
AEA: *N*-arachidonoylethanolamide
CAD: Coronary artery disease
eCB: Endocannabinoid
LC/MRM: Liquid chromatography/multiple reaction monitoring
NSTEMI: Non-ST-segment elevation myocardial infarction
PEA: Palmitoylethanolamide
STEMI: ST-segment elevation myocardial infarction
