# Supplementary figures and images for: Endocannabinoid 2-arachidonoylglycerol is elevated in the coronary circulation during acute coronary syndrome
Source: PLoS One. 2019 Dec 30;14(12):e0227142. doi: 10.1371/journal.pone.0227142 (PMC6936850; doi:10.1371/journal.pone.0227142)

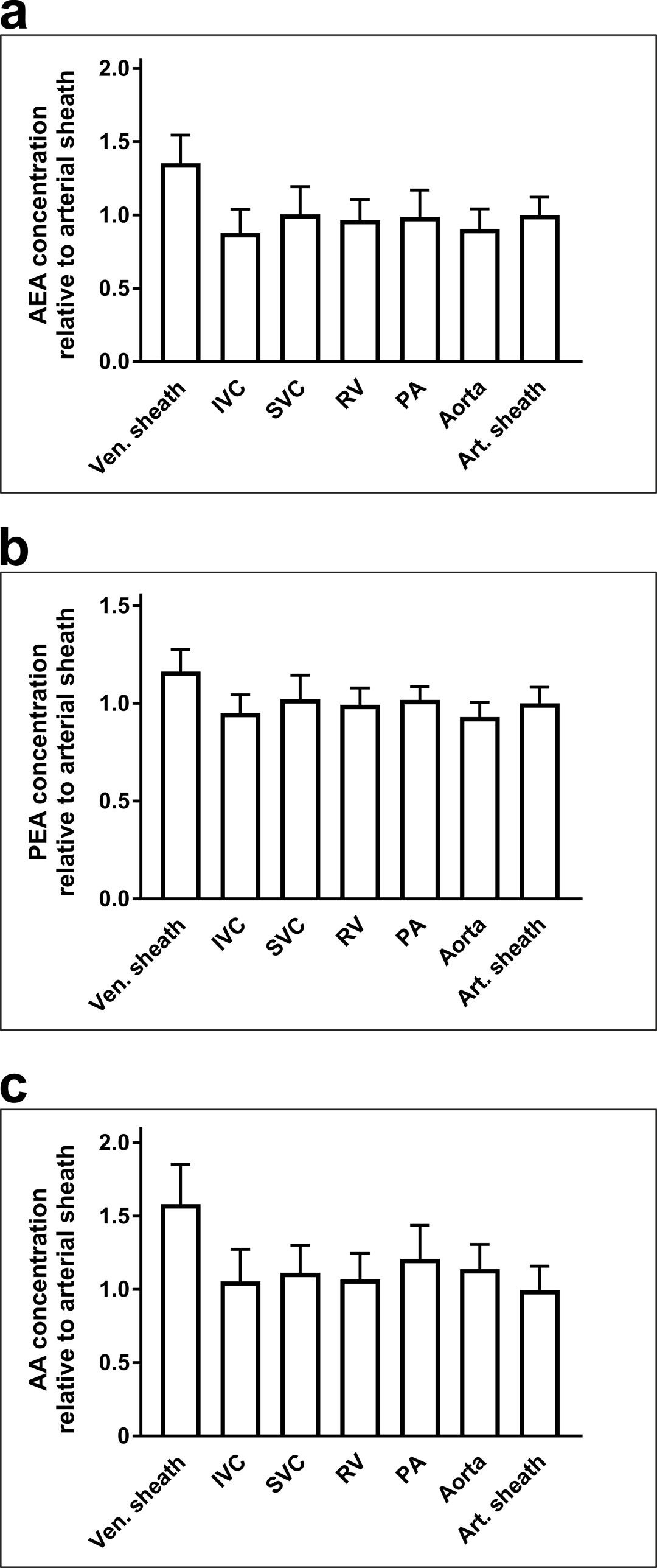

Supplement: S2 Fig — Blood samples were drawn at distinct locations during combined right- and left-heart catheterization. Sites of blood extraction were the venous sheath, the inferior vena cava (IVC), the superior vena cava (SVC), the right ventricle (RV), and the pulmonary artery (PA), as well as the aorta and the arterial sheath. Data are presented as the mean ± standard error of the mean. AA, arachidonic acid; AEA, N-arachidonoylethanolamide; PEA, palmitoylethanolamide. (TIF) [file pone.0227142.s002.tif]
