# Supplementary material for: Endocannabinoid 2-arachidonoylglycerol is elevated in the coronary circulation during acute coronary syndrome
Source: PLoS One. 2019 Dec 30;14(12):e0227142. doi: 10.1371/journal.pone.0227142 (PMC6936850; doi:10.1371/journal.pone.0227142)
Supplement: S1 Table — P-values as indicated. 2-AG, 2-arachidonoylglycerol; AA, arachidonic acid; AEA, N-arachidonoylethanolamide; BMI, body mass index; CK-MB, creatine kinase-muscle/brain; CRP, c-reactive protein; Hb, hemoglobin; HDL, high-density lipoprotein; IL, interleukin; LDL, low-density lipoproetin; PCT, procalcitonin; PEA, palmitoylethanolamide. (DOCX) [file pone.0227142.s003.docx]

**Endocannabinoid 2-arachidonoylglycerol is elevated in the coronary circulation during acute coronary syndrome**

Julian Jehle^1*^, Hanna Goerich^1^, Laura Bindila^2^, Beat Lutz^2^, Georg Nickenig^1^ and Vedat Tiyerili^1^

1. Department of Internal Medicine II Cardiology, Pneumology, Angiology, University Hospital Bonn, Bonn, Germany
2. Institute of Physiological Chemistry, University Medical Center of the Johannes Gutenberg University Mainz, Mainz, Germany

**Supporting Information**

**S1 Table. Pearson’s correlation coefficients (r) of endocannabinoid levels (sheath) with clinical parameters and laboratory findings.**

| **Correlation with** | **2-AG** | **AEA** | **PEA** | **AA** |
| --- | --- | --- | --- | --- |
| Age | r = 0.0259  p = 0.8256 | r = 0.1164  p = 0.3235 | r = 0.0372  p = 0.7653 | r = -0.0208  p = 0.8862 |
| Weight | r = -0.0803  p = 0.5527 | r = 0.2508  p = 0.0647 | r = -0.0337  p = 0.8182 | r = 0.2395  p = 0.0939 |
| Height | r = 0.0683  p = 0.6236 | r = -0.2616  p = 0.0610 | r = -0.1377  p = 0.3559 | r = -0.1442  p = 0.3282 |
| BMI | r = -0.1158  p = 0.4045 | ***r = 0.3703***  ***p = 0.0069*** | r = 0.0460  p = 0.7588 | ***r = 0.3215***  ***p = 0.0259*** |
| Systolic blood pressure | r = -0.1020  p = 0.3841 | r = -0.0165  p = 0.8893 | r = -0.0326  p = 0.7937 | r = -0.0703  p = 0.5687 |
| Diastolic blood pressure | r = 0.0550  p = 0.6394 | ***r = 0.2502***  ***p = 0.0315*** | r = 0.1719  p = 0.1641 | r = 0.0750  p = 0.5434 |
| Heart rate | r = -0.0257  p = 0.8281 | ***r = 0.2553***  ***p = 0.0293*** | ***r = 0.3478***  ***p = 0.0042*** | r = 0.0730  p = 0.5571 |
| Erythrocytes | r = -0.1083  p = 0.3550 | ***r = 0.3045***  ***p = 0.0083*** | r = 0.1926  p = 0.1184 | ***r = 0.2524***  ***p = 0.0379*** |
| Hb | r = -0.1639  p = 0.1600 | ***r = 0.2765***  ***p = 0.0171*** | ***r = 0.2660***  ***p = 0.0296*** | ***r = 0.2596***  ***p = 0.0325*** |
| Hematocrit | r = -0.1592  p = 0.1726 | ***r = 0.3086***  ***p = 0.0075*** | ***r = 0.2867***  ***p = 0.0186*** | r = 0.2271  p = 0.0625 |
| Thrombocytes | r = -0.0218  p = 0.8526 | r = 0.0116  p = 0.9216 | r = -0.1227  p = 0.3224 | r = 0.0707  p = 0.5667 |
| Leukocytes | r = 0.0325  p = 0.7819 | r = -0.0968  p = 0.4122 | r = -0.1090  p = 0.3798 | r = 0.1127  p = 0.3600 |
| Lymphocytes | r = 0.0384  p = 0.7613 | r = -0.1265  p = 0.3191 | r = -0.1859  p = 0.1586 | r = 0.1725  p = 0.1914 |
| Monocytes | r = - 0.0020  p = 0.9876 | r = -0.0320  p = 0.8021 | r = 0.1631  p = 0.2170 | r = 0.0271  p = 0.8387 |
| Neutrophil Granulocytes | r = -0.0653  p = 0.6055 | r = 0.0180  p = 0.8878 | r = 0.0231  p = 0.8623 | r = 0.0343  p = 0.7967 |
| Eosinophil Granulocytes | r = 0.0255  p = 0.8413 | r = -0.1202  p = 0.3482 | r = -0.1207  p = 0.3670 | r = -0.1461  p = 0.2739 |
| Basophil Granulocytes | r = -0.0417  p = 0.7418 | r = -0.0632  p = 0.6199 | r = -0.0061  p = 0.9637 | r = -0.0307  p = 0.8174 |
| CRP | r = 0.0387  p = 0.7538 | r = 0.0604  p = 0.6272 | r = 0.0038  p = 0.9769 | r = -0.2258  p = 0.0802 |
| PCT | r = 0.0214  p = 0.8758 | r = 0.1365  p = 0.3158 | r =0.0224  p = 0.8722 | r = -0.1367  p = 0.3196 |
| IL-6 | ***r = 0.4235***  ***p = 0.0013*** | r = -0.1066  p = 0.4387 | r = 0.0957  p = 0.4911 | r = -0.0865  p = 0.5300 |
| IL-8 | r = -0.1165  p = 0.4060 | r = -0.1140  p = 0.4117 | r = -0.1702  p = 0.2232 | r = -0.0447  p = 0.7482 |
| Complement 3 | r = -0.0670  p = 0.6271 | r = 0.1627  p = 0.2354 | r = -0.1366  p = 0.3247 | r = 0.1582  p = 0.2488 |
| Complement 4 | r = -0.1499  p = 0.2747 | r = 0.1592  p = 0.2457 | r = -0.1309  p = 0.3455 | r = -0.0084  p = 0.9516 |
| HbA1c | r = -0.0854  p = 0.5025 | r = 0.0815  p = 0.5255 | r = -0.0099  p = 0.9406 | r = -0.0347  p = 0.7926 |
| Peak troponin | ***r = 0.4564***  ***p < 0.0001*** | r = 0.1100  p = 0.3507 | r = 0.0350  p = 0.7788 | r = 0.2242  p = 0.0661 |
| Peak CK-MB | r = 0.1049  p = 0.3703 | r = 0.0594  p = 0.6150 | r = 0.2189  p = 0.0751 | r = 0.0414  p = 0.7377 |
| Total cholesterol | r = 0.0050  p =0.6981 | r = 0.1626  p = 0.2029 | ***r = 0.2556***  ***p = 0.0468*** | r = 0.2204  p = 0.0878 |
| LDL | r = 0.0614  p = 0.6056 | r = 0.1848  p = 0.1175 | ***r = 0.2990***  ***p = 0.0147*** | r = 0.1226  p = 0.3269 |
| HDL | r = -0.1347  p = 0.2558 | r = -0.0876  p = 0.4612 | r = 0.1120  p = 0.3706 | r = 0.0667  p = 0.5948 |
| Triglycerides | r = 0.1073  p = 0.3730 | r = 0.0115  p = 0.9243 | r = -0.0663  p = 0.6028 | r = 0.0793  p = 0.5333 |

P-values as indicated. 2-AG, 2-arachidonoylglycerol; AA, arachidonic acid; AEA, *N*-arachidonoylethanolamide; BMI, body mass index; CK-MB, creatine kinase-muscle/brain; CRP, c-reactive protein; Hb, hemoglobin; HDL, high-density lipoprotein; IL, interleukin; LDL, low-density lipoproetin; PCT, procalcitonin; PEA, palmitoylethanolamide.
